# Successful repair of injury to the eyelid, lacrimal passage, and extraocular muscle

**DOI:** 10.3205/oc000041

**Published:** 2016-03-17

**Authors:** Shreya Mehul Shah, Mehul Ashvin Shah, Prerna D. Shah, Kashyap B. Patel

**Affiliations:** 1 Drashti Netralaya, Dahod, Gujarat, India

**Keywords:** lacrimal canalicular injury repair, extraocular muscle rupture repair, lid laceration repair

## Abstract

**Introduction:** Injury is a known cause of monocular blindness. Ocular trauma may affect lacrimal canaliculi and the extraocular muscle. We report this case as it includes injury to lid, lacrimal canaliculi and inferior rectus.

**Case description:** A 25-year-old male presented with an injury caused by a sharp object that resulted in a conjunctival tear, lid tear involving the lacrimal canal, and rupture of the inferior rectus muscle. All of the structures were repaired successfully during a single procedure.

**Conclusion:** An extraocular injury involving the conjunctiva, lid, lacrimal passages, and extraocular muscles can be repaired successfully during a single procedure.

## Introduction

Lid lacerations are common with ocular injuries [1], [2], [3], while an association with lacrimal passage injuries is less common [1]. The traumatic rupture of extraocular muscles is rare. We observed all three in one case and successfully repaired the lacrimal canal, inferior rectus muscle, and conjunctiva all during a single procedure. The postoperative follow-up has been uneventful for 4 years.

## Case description

**Figure 1:**
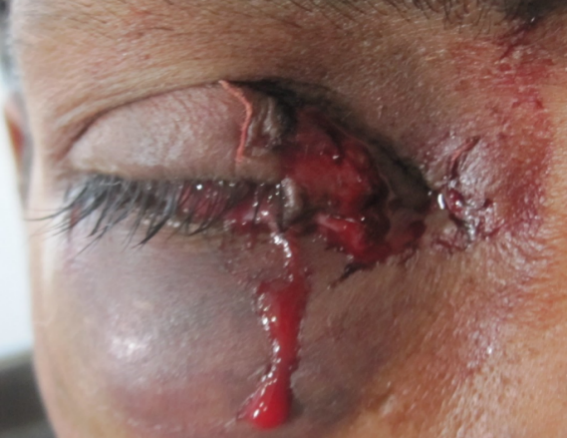
State at presentation

A 25-year-old male presented to our outpatient department following an injury to the lid of his left eye (OS), caused by a sharp iron object, that occurred while working without taking safety measures (Figure 1 (Fig. 1)).

**Figure 2:**
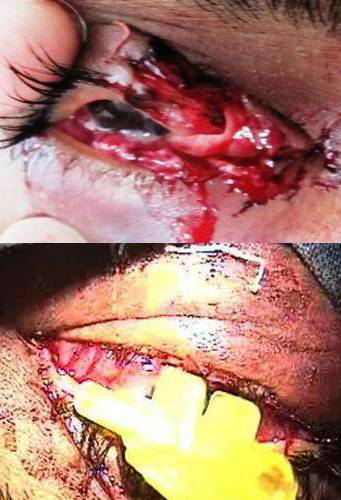
Proximal and distal ends of lacrimal canaliculi

**Figure 3:**
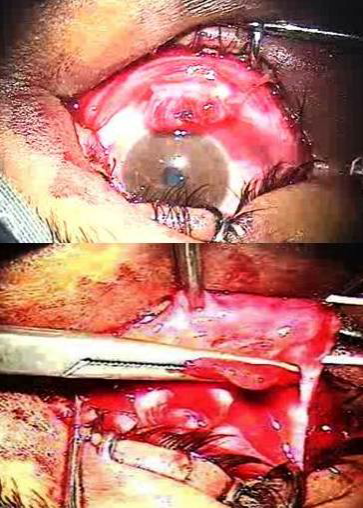
Proximal and distal ends of inferior rectus muscle

On examination, he had an injury to the lower lid involving the lacrimal canal (Figure 2 (Fig. 2)), a tear in the conjunctiva, and a ruptured inferior rectus muscle (Figure 3 (Fig. 3)). He also had hypertropia of the OS and absent inferior movement in the affected eye. 

The eyelid was repaired surgically in layers. The lacrimal canal was repaired using the Teflon sleeve of a 22G Intracath (Figure 2 (Fig. 2)), which was left in place for 6 weeks. We located the proximal end of the canal and confirmed it by syringing. We found the proximal end of the inferior rectus muscle and sutured it to the distal end, achieving proper strength, as detected by orthophoria in the primary position and inferior movements at the end of the repair (Figure 3 (Fig. 3)).

**Figure 4:**
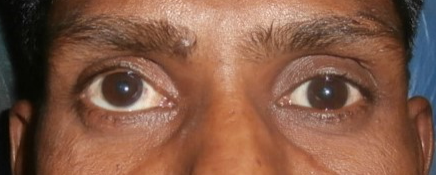
Presentation with hypertropia after 3 years of repair

The patient complained of diplopia in the early postoperative period. This was evaluated with diplopia charts and the patient improved slowly, both subjectively and objectively. Patient presented to us again with complaint of diplopia after 3 years, on examination we detected right eye hypertropia (Figure 4 (Fig. 4)), inferior rectus resection was done and became orthophoria.

**Figure 5:**
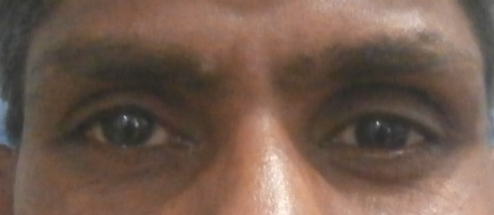
Final image after inferior rectus resection

The patient achieved orthophoria and full movement in inferior gaze (Figure 5 (Fig. 5)).

## Discussion

Lid lacerations and repair have been reported, with or without involvement of the lacrimal canal. However, a laceration involving the lid, lacrimal canal, and extraocular muscle is unique and the successful repair of all components during a single procedure has not been reported.

Lacrimal canal lacerations are common in ophthalmic practice, but direct injury to the canal is reported only in 16% to 54% [1], [4] of cases. Variable success rates have been reported for lacrimal canal repair using different techniques, including a pig tail needle [2] or silicon tube for intubation [2], [3], or a mini-monoka [3].

There are few reports on the rupture of extraocular muscles [4], [5], [6], [7]. Rupture of the inferior rectus has been reported in three studies [5], [7]. Harish et al. reported rupture of the superior oblique [4], while Sari et al. reported the rupture of three rectus muscles in blunt trauma [5].

Our case is a unique case involving lid, lacrimal canaliculi and extraocular muscle. 

## Conclusion

An extraocular injury involving the conjunctiva, lid, lacrimal passages, and extraocular muscles can be repaired successfully during a single procedure.

## Notes

### Competing interests

The authors declare that they have no competing interests.

### Ethics

Ethical committee and patient consent obtained for this case report.
